# The Bright Side of COVID-19: Integrated Art-based and Virtual Learning in Medical Education

**DOI:** 10.30699/ijp.2021.521015.2548

**Published:** 2021-05-09

**Authors:** Afsaneh Yakhforoshha, Fatemeh SamieeRad

**Affiliations:** 1 *Education Development Center, Faculty of Medical School, Qazvin University of Medical Sciences, Qazvin, Iran*; 2 *Department of Pathology, Faculty of Medical School, Qazvin University of Medical Sciences, Qazvin, Iran*

**Dear Editor**

In many crisis situations like the COVID-19 pandemic, medical education has not been spared. Extensive efforts will be exerted to develop competencies in health care, which are professionalized as well as sufficiently flexible. In order to develop this adaptive expertise, trainees should be engaged in the learning process. Several programs offer art-based teaching as an innovative andragogy tool for making more realistic and experiential learning environments and thereby, helping students develop various competences such as critical thinking, creativity, reflection, observational skills, etc. (1, 2).

On the other hand, in the COVID-19 era, instructors who teach fundamental hands-on courses will need to use technology to transfer education to a virtual learning environment. Therefore, Iran’s Virtual University of Medical Sciences (VUMS) has provided National Learning Management System (NAVID) for all medical universities in Iran. Therefore, Qazvin University of Medical Sciences has adequate infrastructures for virtual learning.

In response to a call for practical pathology teaching to reflect the clinical context, there has been a shift to engage medical students in learning opportunities. We created a visual art-based pathology course with a combination of synchronous and asynchronous activities. In terms of asynchronous activities, we used NAVID for provision of educational contents, assignment, student evaluations and a discussion room. 

In terms of synchronous activities, using adobe connect, we facilitated observational exercises using visual thinking strategies (VTS) to assist students in empowering critical thinking and observation skills (interpreting visual cues to recognize patterns) (3).

In the pathology course, medical students interacted with a variety of course content and engaged with their classmates and educator. To keep students engaged with the content, the course content was organized in different formats such as text-based readings, glass slide images which were prepared using a camera microscope (Olympus BX53, Japan), in 40, 100 and 400 magnifications, and video lessons presenting different learning methods. In addition, various assignments and art-based activities and discussion boards were designed to offer opportunities for students to be engaged. 

For learner–learner and learner–instructor interactions, each student actively draw at least a single pathologic image (painting) for each pathology unit encountered over the term without restrictions. In order to evaluate students’ learning, educator defined some formative assessments to monitor their progress during the course in the adobe connect collaborate sessions. These sessions comprise observational activity using visual thinking strategies (VTS) (4). Before being enrolled in the study, informed consent was obtained from the students under the supervision of the Ethics Committee of Kosar educational Hospital.

During this experience, students presented and shared their own artwork using Adobe Connect. Then, the teacher provided the students with an opportunity to pay attention to visual cues of paintings and facilitated group discussion through the following stages:

- Asking three questions to help students look carefully and engage with their peers and teacher; “what do you see in this painting?’ “What do you see and what makes you say that?” and “What else can you find?” (5).
- Encouraging students to express their opinions about the art-works

- Making sure that comments from all students are acknowledged by the instructor
- Repeating observations and emphasizing on features of the creative art displayed
- Having group discussions on students’ observations

To obtain medical students’ opinion about the course, we designed a semi-structured interview with medical students in Qazvin Medical School, Iran. They said that art-based teaching with virtual learning made a valuable contribution to the pathology course and their appreciation of paintings helped them develop their learning and assisted them to facilitate the diagnosis of medical pathologies.

Therefore, in a novel threatening situation to human health, continuation of education has been provided for house-bound medical students, with innovative learning modalities such as various virtual tools and visual arts. In spite of being responsible for the crisis, COVID-19 epidemic will also be viewed as an opportunity in delivering medical education.

Our results illustrated the important role of educational strategies in medical curriculum during crisis situations. In 1968, Malcolm Knowles introduced the theory on Andragogy focusing on the learning strategies of adults.

He organized his Andragogical Model around multi presumption (i.e. self-directing in the learning process, enjoying learning through experience, being motivated by internal factors, being interested in problem-centered learning, being responsible for the learning process and integrating learning into the real world.

Self-directed learners bring a wealth of experience to the educational setting; adults enter educational settings ready to learn. They are problem-centered in their learning and best motivated by internal factors (6).

This approach to learning is in accordance with the first of six strategies of Harden SPICES model for curriculum development, which is a student-centered and not a teacher-centered approach (S) (7, 8). In conclusion, the results from medical students and the Knowles’ Andragogical Model as a theoretical lens, is expected to help medical education experts with emphasizing on a student-centered approach in medical education curriculum in order to prepare future physicians for meeting unexpected healthcare crisis demands.

**Fig. 1:**
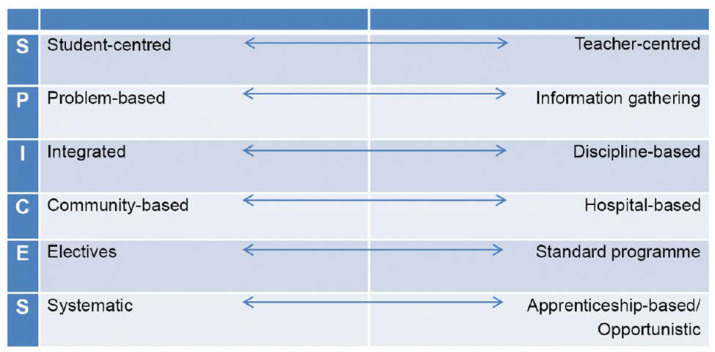
The SPICES Model of Educational Strategies
